# Communication as a powerful tool in the treatment of war veterans with chronic posttraumatic stress disorder and chronic pain

**DOI:** 10.3325/cmj.2019.60.479

**Published:** 2019-10

**Authors:** Neda Pjevač, Marijana Braš, Veljko Đorđević, Nada Pjevač

**Affiliations:** 1 Department of Medical Statistics, Epidemiology and Medical Informatics, University of Zagreb, School of Medicine, Andrija Štampar School of Public Health, Zagreb, Croatia *npjevac@mef.hr*; 2 Center for Palliative Medicine, Medical Ethics and Communication Skills, University of Zagreb School of Medicine, Zagreb, Croatia; 3 Department of Psychiatry and Psychological Medicine, Clinical Hospital Centre Zagreb, Zagreb, Croatia; 4 Medical Center Zagreb “Centar,” Zagreb, Croatia

The latest revision of the Diagnostic and Statistical Manual of Mental Disorders (DSM-5, 2013) included a number of evidence-based changes to the diagnostic criteria for posttraumatic stress disorder (PTSD) (1). PTSD is now classified in a new category, Trauma-and Stressor-Related Disorders. The rate of lifetime PTSD is two to four times higher among war veterans than in the general population (2). Chronic PTSD is associated with various somatic illnesses, from autoimmune to cardiovascular, and especially with chronic pain (3). During the Croatian Homeland War (1991-1995) and its aftermath, a considerable number of people in Croatia were exposed to long-term and intense traumatic experiences. In this essay we would like to emphasize the importance of adequate communication in the treatment of war veterans with chronic PTSD and chronic pain, especially since communication has an important role in treatment outcomes.

## Chronic posttraumatic stress disorder and chronic pain

Chronic pain is a psychosomatic disorder with physical, mental, social, and spiritual components, as well as one of the best clinical examples of the mind-body relationship. In war veterans, PTSD and chronic pain show high comorbidity (4-7), a relationship that can be explained by PTSD-associated alterations to the hypothalamic-pituitary-adrenal axis and sympatho-adrenal medullary-axis, with implications on neuroendocrine and immune functioning (4,5). Individuals with PTSD and chronic pain have more severe symptom presentation, higher rates of health care utilization, more pain-related disability, higher pain ratings, increased functional impairment, and increased health care costs (8). When it comes to the treatment of these individuals, a very important modality is person-centered approach, with a special emphasis on “rational polypharmacy.” Although the best option for patients with PTSD and chronic pain is interdisciplinary treatment, Croatian outpatient pain units comprise mostly anesthesiologists, neurologists, or psychiatrists alone.

## The importance of person-centered communication

The current model of collaborative partnership between the physician and patient emphasizes an educated patient as well as physician’s educational and advocacy role (9). This model highlights communication as one of the most important clinical skills. Communications skills are especially important when it comes to overcoming communication challenges, such as breaking of bad news, recognizing patients’ acute and chronic emotional reactions, organizing family meetings, communicating risks and prognosis, communicating end-of-life issues, and communicating in an interdisciplinary team. Francis W. Peabody said: “One of the essential qualities of the clinician is interest in humanity, for the secret of the care of the patient is in caring for the patient.” (10) Communication skills training is now widely accepted as an essential component of health education (11), as well as of burn-out prevention among health professionals. Although in the Skill Laboratory of the School of Medicine University of Zagreb, we started teaching communication skills for health professionals as early as at the beginning of Croatian Homeland War, much progress has happened in the last ten years. In September 2010, the Centre for Palliative Medicine, Medical Ethics, and Communication Skills (CEPAMET) was founded as an organizational unit of the University of Zagreb School of Medicine. CEPAMET provides communication skills education for medical students, health care professionals, and the general public (12). In 2011, an elective course in communication skills for medical students was introduced, with a special emphasis on experiential learning as a teaching method. A few years later, on the initiative of the dean, a mandatory six-year longitudinal course, Fundamentals of Medical Skills, was introduced, with an aim to provide training in both clinical and communication skills. Communication skills teaching is also a part of the post-graduate competencies teaching for all medical residency programs in Croatia (13). In all these courses, Calgary-Cambridge model of medical interview is used as a dominant model. Our communication skills teaching integrates person-centered medicine and people-centered health care (14,15). Only a small amount of teaching is organized in the form of traditional lectures (11), while greater emphasis is placed on specific experiential teaching methods, where students and professionals get a chance to practice with real-life and simulated patients using video cameras and scales (12).

CEPAMET has undertaken various research projects on communication skills issues. Also, we promote the Zagreb model of person-centered medical interview, which is focused on patient’s quality of life in the context of health and disease (16). Another very important component of communication in medicine is art, which can be used as a therapeutic technique but also as powerful teaching method, especially when simulated patients are used (17).

## Communication with war veterans with chronic pain and posttraumatic stress disorder

Professionals working with the Croatian Homeland War veterans and their families face numerous communication challenges. These challenges could be overcome by providing the professionals with continuous professional education, primarily through experiential teaching methods. Continuous education is crucial since our recent study suggested that, although they are aware of the importance of communication, physicians believe that during their studies they did not receive appropriate education about communication skills (18). It is not only physicians who should be educated, but also all other members of an interdisciplinary team (nurses, psychologists, social workers, educators, nutritionists, physical therapists, occupational therapists, and students). There is also a need for interdisciplinary counseling in analyzing physical, psychological, social, and spiritual aspects of war veterans’ health. Better and more comprehensive communication might also be achieved through encouraging scientific, professional, educational, and public health activities dedicated to the health status of the veteran population.

The dignity and quality of life of this population is further undermined by stigmatization. This problem might be tackled by fostering communication with the general public, which often holds negative prejudice and lacks knowledge on veterans’ health. We are proud that we designed special modules dedicated to teaching communication with patients with war-related PTSD and comorbid chronic pain, both in undergraduate and postgraduate education as well as in various post-graduate continuing education workshops. Also, we use art to educate the general public about these comorbidities, especially through the projects Communication against Pain and On the Front Line of Health. War veterans are an important part of our community, and the worst approach would be to ignore their suffering. This is why it is important to act urgently and continue with the development of educational programs by emphasizing person-centered medicine and the culture of health (19).

## Conclusion

Since the overall health of war veterans in Croatia remains a major problem, it is necessary to develop person-centered interdisciplinary health care programs for this population, while placing special emphasis on their quality of life. To achieve this, we have to recognize the interconnection of the veterans’ psychological, physical, social, and spiritual health. Croatia is developing a national strategy on the protection and promotion of health in this population. Therefore, institutions and organizations dealing with the health of war veterans need to cooperate better and perform a continuous and detailed epidemiological analysis related to PTSD morbidity and mortality. However, the first and most important step in the treatment of war veterans is person-centered communication. Communication is an integral part of any relationship with veterans and their families, and represents the key to the treatment success. *Homo homini remedium*!
